# Dimeric magnetic dumbbell nanoparticles with selective immobilization of chromophores for improved tumor theranostics

**DOI:** 10.1038/s41598-026-40586-4

**Published:** 2026-03-05

**Authors:** Iuliia Chudosai, Petr Ostroverkhov, Ekaterina Plotnikova, Kseniya Stepanova, Nelly Chmelyuk, Elizaveta Ivanova, Mihail A. Grin, Olga Fedorova, Natalia Klyachko, Vladimir P. Chekhonin, Maxim Abakumov

**Affiliations:** 1 Department of Chemistry, Lomonosov Moscow State University, Moscow, 119991 Russia; 2 Pirogov Russian National Research Medical University, Ostrovitianov Str. 1, Moscow, 117997 Russia; 3 National Research Technological University “MISIS”, Biomedical Nanomaterials, Leninskiy Prospekt 4, Moscow, 119049 Russia; 4 Russian Technological University (MIREA), 86 Vernadsky Avenue, Moscow, 119571 Russia; 5 National Medical Research Radiological Centre of the Ministry of Health of the Russian Federation, P.A. Hertsen Moscow Oncology Research Institute, Moscow, 125284 Russia; 6 A. N. Nesmeyanov Institute of Organoelement Compounds, Russian Academy of Sciences, Moscow, 119334 Russian Federation

**Keywords:** Nanoparticles, FRET effect, PDT, Fluorescent diagnostics, Cancer theranostics, Photosensitizer, Biological techniques, Biotechnology, Cancer, Chemistry, Materials science, Nanoscience and technology

## Abstract

Recently, the development of dimeric nanoparticles has attracted much attention from researchers, since these materials make it possible to combine various properties and functions in one system. Dumbbell nanoparticles enable the integration of various components to achieve dual functionality, for example imaging and therapy. In our work, we showed that «dumbbell» type magnetite-gold dimeric nanoparticles could be used for combining Cy5 fluorophore and bacteriopheophorbide based photosensitizer for the joint use in fluorescent diagnostics and photodynamic therapy, allowing to avoid the FRET effect. Surface modification of magnetite surface by DOPAC and PEG allows selective photosensitizer binding, whereas disulfide derivative of Cy5 fluorophore was conjugated to gold surface, providing sterical separation of chromophores, thus minimizing FRET effect, and providing colloidal stability in water solutions. Nanoparticles, containing a pair of chromophores can be internalized by CT26 colon cancer cells and fluorophore and photosensitizer fluorescent signals were colocalized in cells cytoplasm and perinuclear area. Cytotoxicity tests have shown no significant negative effects on CT26 cells in vitro at concentrations up to 100 µg/mL. Finally, light induced toxicity in time dependent manner was shown. Proposed approach opens new opportunities for simultaneous fluorescent diagnostics and photodynamic therapy of tumors for photosensitizers with low intrinsic fluorescence.

**Supplementary Information:**

The online version contains supplementary material available at 10.1038/s41598-026-40586-4.

## Introduction

Cancer therapy is the acute problem of scientists and doctors all over the world. On average, ten million people in the world are diagnosed with cancer every year with the frequency of cases increased annually. This increase associated both with the improvement of diagnostic methods and with the influence of factors provoking such diseases.

Among the different approaches in cancer treatment nanotechnology based theranostics platforms show promising results^1–5^. A lot of attention is paid to design of new systems in which various components are integrated to achieve both imaging and therapy^6–11^.

One of the successful methods of cancer therapy is photodynamic therapy (PDT)^12^. PDT is a treatment strategy that uses a compound called a photosensitizer and light radiation. When photosensitizers are exposed to light of a specific wavelength, they produce reactive oxygen species (ROS) that are toxic to cells. PDT is low invasive procedure with such advantages as relatively low toxicity due to only local light irradiation, as well as the possibility of combination with other methods of cancer treatment. As with all methods, there are disadvantages: (1) low penetration of patient tissues with light at visible region, requiring use of photosensitizer (PS) with extinction/emission wavelength shifted to IR region, where tissues are most transparent for light; (2) time of PS accumulation in tumor have to be carefully evaluated to estimate starting point of light irradiation, when PS concentration in tumor reaches its maximum^13^. Luckily most of the PS show not only generation of ROS, but also are by themselves fluorophores allowing simultaneous detection and therapy using the same molecule. On the other hand, when excited by light, two mutually exclusive options for “using” the PS energy arise - the ability to fluoresce and ability to produce ROS. Also, low values of the Stokes shift of currently used PS are limiting the ability to separate fluorescent signal from PS and reflected by tissues signal from excitation beam.

Based on the foregoing, to detect a PS, one of the approaches is to combine a PS and a fluorescent label, a fluorophore (FP), in one platform to provide PDT and PS detection (fluorescence diagnostics, FD). However, the main problem in this approach is the nonradiative energy transfer or “the FRET problem” (Förster nonradiative energy transfer). This problem arises from the ability of energy nonradiatively being transferred from FP to PS, thus leading to quenching of FP and not allowing the FP to be detected, or the energy transfer from PS to FP, leading to situation when the energy flows out from PS, thus reducing ROS formation and subsequently PDT efficacy. There are two options for solving this problem, namely, to select a pair of PS and FP with nonoverlapping excitation/emission wavelength, thus bypassing the FRET mechanism, or by increasing distance between a pair of chromophores (PS and FP), separating them in space thus not allowing FRET to occur. Many molecules are known in the scientific literature to be used as FP and PS, but evaluation of the absorption and fluorescence spectra of chromophores, shows that it is impossible to select such a pair of FP and PS without the FRET mechanism, since the spectra of chromophores are located in the visible radiation range (from ~ 300 nm to ~ 800 nm) and have overlap zones. In other words, any pair of chromophores will have a FRET effect, to a greater or lesser extent. The promising solution to the “FRET problem” can be carried out by stereometric separation of two chromophores (FP and PS) in space in order to avoid the FRET effect.

One of the examples of the possibility of separation of two chromophores in space was proposed using two flexible oligo (ethylene glycol) linkers of different lengths. Conjugation of 4-pyrazolinyl-1,8-naphthalimide and propargyl-15,17-dimethoxy-13-amide and bacteriochlorin led to the creation of a dual-functional system including a PS and a fluorescence imaging agent. This type of fluorescent dyes exhibited high Stokes shifts, good photostability, and in vitro imaging capability^14^. However, there are disadvantages in the above system, such as the system exhibiting a higher intensity of emission from the naphthylamide unit, which is explained by an increase in the efficiency of resonant energy transfer between chromophores. The fluorescence of naphthylamide in the studied conjugates greatly increased due to the resonant energy transfer to the acceptor fragment (FP). Moreover, visualization was possible only after high power irradiation, which causes photodegradation of the bacteriochlorin chromophore^15^.

One of the most interesting objects from the point of view of application in biomedicine is hybrid structures based on magnetic nanoparticles (NPs) and noble metal^16^. The use of the magnetic component makes it possible to use the MRI method and cancer hyperthermia as an additional option for influencing cancer cells^17–20^. One the other hand having both magnetite and gold surfaces provides a unique opportunity for selective modification of them. This opportunity allows immobilization of different molecules with their separation in space, but still being connected to one NP. In case of PS an FP molecules such space separation might allow to overcome existing “FRET problem”, thus allowing simultaneous imaging and therapy with high efficiency.

Based on the foregoing, in this article we have for the first time described and characterized a platform based on dumbbell-shaped magnetite-gold NPs immobilized with PS (bacteriopheophorbid series, φ^1^О_2_=0.79) and FP (cyanine series, disulfide derivative Cy5, φ_fl._ = 0.28), which allows the use of PDT and FD simultaneously (without energy transfer via the FRET mechanism).

## Methods

### Materials

The following reagents were used to obtain, modify, and functionalize dimer magnetite-gold NPs: oleic acid (C_12_H_3_4O_2_, 90%, Sigma-Aldrich, USA), oleylamine (C_18_H_35_NH_2_, 70%, Sigma-Aldrich, USA), 1-octadecene (C_18_H_36_, 90%, Sigma Aldrich), iron pentacarbonyl (Fe(CO)_5_, 99.9%, Sigma Aldrich), hydrosauric acid (III) trihydrate (HAuCl_4_, 99.9%, Sigma Aldrich), acetone (C_3_H_6_O, Re -akhim), hexane (C_6_H_14_, Reakhim), 3,4-dihydroxyphenylacetic acid (DOPAC, Sigma-Aldrich, USA), sodium hydroxide (NaOH, 95%, BioPharmCombinat, Russia), methyl alcohol (CH_3_OH, 95%, BioPharmCombinat, Russia), N-hydroxysuccinimide (NHS, 98%, Sigma-Aldrich, USA), 1-ethyl-3-(3-dimethylaminopropyl)carbodiimide (EDC, 98%, Sigma-Aldrich, USA), poly(ethylene glycol) 2 -aminoethyl ester of acetic acid (Mn 1.100, Sigma Aldrich, USA), chloroform (CHCl_3_, Component-Reaktiv, Russia), 3-(2-pyridyl)−5,6-diphenyl-1,2,4 monosodium salt hydrate -triazine-p, p’-disulfonic acid (ferrozine, C_20_H_13_N_4_NaO_6_S_2_*xH2O, 97%, Sigma Aldrich), concentrated hydrochloric acid (HCl, 36%, Reakhim), Amicon Ultra 30 kDa centrifuge filters. For the preparation of all solutions in the processes of synthesis and analysis, we used deionized distilled (DI) water prepared in a Milli-Q-RO4 system (Millipore). 131-(4-aminobutylcarbamoyl)bacteriochlorin methyl ester (synthesized by colleagues at MIREA), sulfocyanine-5 NHS ester (Cy5, Lumiprobe, Russia), cystamine dihydrochloride (96%, Sigma-Aldrich, USA), tablets for the preparation of sodium phosphate buffer solution (1xPBS, Sigma-Aldrich, USA), deionized distilled (DI) water, Amicon Ultra 10 and 30 kDa centrifuge filters.

All obtained volumes of substances were filtered through a Millipore membrane filter with a pore size of 0.45 μm. Next, the resulting samples were diluted in 24-well plates (Corning, USA) in cell culture medium. The final concentration of substances varied from 4000 ng/mL to 20 ng/mL.

### Synthesis of the compounds

#### Synthesis of hybrid dumbbell-shaped Fe_3_O_4_-Au NPs

The synthesis of hybrid dumbbell-shaped Fe_3_O_4_-Au NPs was carried out by the combined thermal decomposition of Fe(CO)_5_ and HAuCl_4_according to the procedure taken from with some modifications^21^. 20 mL of 1-octadecene, 2 mL of oleylamine, and 1.9 mL of oleic acid were mixed in a 250 mL three-neck round-bottom flask with a thermometer and an argon/nitrogen gas tube. The resulting mixture was heated to 120 °C in an intense flow of inert gas with stirring for 30 min. Next, a system with a reflux condenser was installed, the gas flow was reduced, and 1.9 mL of Fe(CO)_5_ was injected with a syringe. After 3 min, a mixture containing 40 mg of HAuCl_4_·3H_2_O (as a precursor of Au NPs), 0.5 mL of oleylamine and 5 mL of 1-octadecene was introduced, which was previously kept in an inert gas atmosphere for 7–10 min. The reaction mixture was heated at a rate of 3 °C/min to boiling temperature (300–320 °C) and kept at this temperature for 45 min. Next, the solution in the flask was cooled with stirring to room temperature and oxidized with atmospheric oxygen for an h. To purify NPs from excess 1-octadecene, oleic acid, and oleylamine, the resulting solution was distributed in 2 mL into each tube, resuspended in 13 mL of acetone, and centrifuged at a speed of 6000 rpm for 20 min., precipitate was separated by a magnet. This procedure was repeated 4 times until a clear supernatant was obtained. The purified NPs were resuspended in 15 mL of acetone.

#### DOPAC coating of Fe_3_O_4_-Au NPs (NP/DOPAC)

To obtain stable aqueous solutions of magnetite-gold NPs, we chose the method of coating them with 3,4-dihydroxyphenylacetic acid (DOPAC) (Sup. Figure 1). Firstly, a mixture consisting of 51 mg of DOPAC, 24 mg of sodium hydroxide and 10 mL of methanol was prepared. The resulting solution was stirred for 10 min. A mixture of 7 mL of hexane and 3 mL of NPs (C_Fe_ = 1 mg/mL) was added to the resulting solution, after which it was mixed in a water bath at 50 ᵒС for 6–8 h. The mixture was then cooled with continuous stirring overnight.

After that, NP/DOPAC were transferred into centrifuge tubes and precipitated at 6000 rpm for 20 min. The precipitate was removed with a magnet, and then the NPs were dispersed in distilled water and purified by repeated centrifugation in centrifuge filters (30 kDa) at 6000 rpm for 7 min., until supernatant was colorless.

#### PEG coating of NP/DOPAC (NP/DOPAC/PEG)

The NPs were additionally stabilized with polyethylene glycol (PEG) (Sup. Figure 2). 1 mL of NP/DOPAC (C_Fe_ = 1 mg/mL) were mixed with 8 µL (10 mg/mL in H_2_O) of N-hydroxysuccinimide (NHS) and 12 µL (10 mg/mL in H_2_O)) of 1-ethyl-3-(3-dimethylaminopropyl) carbodiimide (EDC). The mixture was stirred for 15–20 min., on a shaker. 100 µL of aminocarboxypolyethylene glycol (PEG, C_PEG_ =100 µg/mL) were added and stirred on a shaker overnight. Next, the excess of the PEG was purified on a centrifuge filter (30 kDa).

#### Characteristics of a FRET pair of chromophores

The pair of chromophores used is a FP of the cyanine series Cy5 and a PS of the bacteriopheophorbide series. Synthetic Cy5 is one of the most popular and commercially available far-red light dyes used in biomedical diagnostics, since it is characterized by high molar absorption coefficient (ε ~ 250000 M^−1^cm^− 1^at 650 nm) and φF = 0.28^22,23^. The sulfonated form of Cy5 (*Sup. Figure 3a*) has improved solubility in water. Sulfo groups create a steric hindrance that prevents the attack of singlet oxygen, which increases the photostability of the dye, in addition, an increase in the number of sulfo groups increases φF, which may be due to the electron-withdrawing effect of sulfo groups on indolenine nitrogen through an inductive mechanism^22,24,25^. The absorption and fluorescence spectra of Cy5 (*Sup. Figure 3b*,* Sup. Figure 3c*), taken in DMSO, have a clearly defined shoulder, which is associated with vibrational transitions^22^. Cy5 has an absorption maximum in the region of the so-called phototherapeutic window, which allows light to penetrate tissue to a depth of 0.9 cm, and fluoresces in the red region of the spectrum, where biological samples have a low background autofluorescence signal. The Stokes shift was 20 nm (absorption ~ 650 nm, emission ~ 670 nm)^26^.

The most promising PSs that absorb light in the optimal wavelength range for PDT are natural and synthetic bacteriochlorins, when used, light penetrates the tissue to a depth of 0.8–1.0 cm^27^. A bacteriochlorin derivative (*Sup. Figure 4a*), synthesized from bacteriopheophorbide, was used as a therapeutic agent. The opening of the exocycle in the initial bacteriopheophorbide increases the hydrophilicity of the resulting PS, which is an important factor, since the main problem of natural and synthetic bacteriochlorins is high hydrophobicity. The spectral characteristics of the PS were measured in DMSO (*Sup. Figure 4b*,* Sup. Figure 4c*). The absorption spectrum has maxima at 350, 520 and 750 nm. When the PS is irradiated with an activation wavelength of 750 nm (ε ~ ​​29600 M^− 1^ cm^− 1^) at a dose of 10 J/cm^2^, the decrease in fluorescence does not exceed 20% while maintaining the appearance of the spectrum, which indicates the photostability of the PS used^28,29^.

#### Synthesis of NP/PS

1 mL of NP/DOPAC/PEG (C_Fe_ = 1 mg/mL) were mixed with 8 µL (10 mg/mL in H_2_O) of N-hydroxysuccinimide (NHS) and 12 µL (10 mg/mL in H_2_O)) of 1-ethyl-3-(3-dimethylaminopropyl) carbodiimide (EDC). The mixture was stirred for 15–20 min., on a shaker. A solution of NHS/EDC activated NP/DOPAC/PEG (С_Fe_ = 1 mg/mL) was added dropwise to a solution of PS in DMSO (0.5 mg/mL) to result in the solvent ratio of DMSO/H_2_O = 30/70 (Sup. Figure 5). The system was left to mix on a shaker overnight. Excess of hydrophobic unbound PS was removed by repeated centrifugation at a speed of 3000 rpm (RCF 1710 g) on ​​centrifuge filters (Millipore Amicon Ultra-4, 30 kDa) for 5 min., with the addition of a mixture of DMSO/H_2_O = 30/70 until the supernatant was transparent, after which was centrifuged twice with the addition of distilled H_2_O to remove DMSO.

#### Synthesis of NP/FP

NPs covalently linked to the dye were prepared by conjugating NP/DOPAC/PEG to a disulfide derivative of Cy5. 0.1 mg of sulfo NHS ester Cy5 in 100 µl of DI H_2_O was added to 0.02 mg of cystamine dihydrochloride in 900 µl of 1xPBS in an Eppendorf tube (pH 8.3–8.5), vortexed and incubated on a shaker at room temperature for 4 h. The resulting disulfide derivative of Cy5 was mixed with 1 mL of NP/DOPAC/PEG solution (C_Fe_ = 1 mg/mL) on a shaker overnight at room temperature. Excess of the dye was removed by repeated centrifugation using centirfuge filters (30 kDa). To confirm complete removal of unbound dye, an aliquot of Fe_3_O_4_-Au/FP was passed through Amicron Ultra-4 (100 kDa) centrifugal filters (Sup. Figure 6).

#### Synthesis of NP/PS/FP

Synthesis of the NP/PS/FP ((Sup. Figure 7) platform was carried out by covalent conjugation on the gold surface to the NP/PS system according to the method of section «Synthesis of NP/FP» (Sup. Figure 6).

## NP/PS, NP/FP and NP/PS/FP characterization

### Registration of spectra

Absorption and fluorescence spectra were obtained on a multifunction analyzer Thermo Scientific Varioskan LUX Multimode Microplate Reader (USA). The spectra were recorded at room temperature in quartz cuvettes (0.4 × 1.0 cm) with an optical path length of 1 cm (spectral slit width 1 nm). The absorption spectra of PS and FP (DMSO solvent) were recorded in the range of 300 ± 900 nm. The fluorescence spectra of PS were recorded in the range 550 ± 900 nm (excitation wavelength 530 nm) and FP in the range 670 ± 900 nm (excitation wavelength 650 nm). The absorption spectra of NP/PS, NP/FP and NP/PS/FP in the were recorded in the range of 300 ± 900 nm.

### Transmission electron microscopy

NP were examined by JEOL JEM-1400 transmission electron microscope operated at 120 kV acceleration voltage. Overview images were taken in conventional bright-field TEM mode.

### XRD analysis

The crystal structure of the samples was studied by X-ray phase analysis (XRD) using a Rigaku Ultima IV diffractometer with Co Kα radiation. Data collection was carried out at diffraction angle values of 2θ = 30–120° with a scanning speed of 0.1° per step and 3 s per point. The results were processed using the software package: OUTSET and PHAN for qualitative phase analysis, SPECTRUM and PHAN for quantitative phase analysis.

### Static magnetic properties of NPs (hysteresis loops, temperature dependence of magnetization)

It was studied on a vibromagnetometer in a Quantum Design PPMS DynaCool system in synthetic capsules containing NP powder or their solution (1 mg · ml^− 1^ in DI H_2_O/1xPBS).

### Dynamic light scattering

The hydrodynamic size and ζ-potential of NPs were determined by dynamic light scattering (DLS) using a Zetasizer Nano ZS analyzer (Malvern Instruments). Dilute aqueous colloidal solutions of samples (С_Fe_ in the range of 0.1–0.3 mg/mL) were measured in backscattering mode at 173 °C and a temperature of 25 °C. The average NP hydrodynamic diameter, surface charge, and standard deviations were obtained from three experiments with 13 measurements for each sample.

### Iron content quantification

Quantification of Fe in NP samples was carried out by a colorimetric method using a FerroZine test and a standard calibration curve. To do this, 20 µl of NP solution was dissolved in 80 µl of concentrated HCl and diluted in 10 times DI H_2_O. To 400 µl of the resulting NP solution, 200 µl of DI H_2_O and 40 µl of ferrozine test were added. Next, 300 µl of the prepared solution was added to 2 wells 96-well plate and analyzed the light absorption intensity at λ = 560 nm on a Thermo Scientific Multiskan GO spectrophotometer.

### Characteristics of in vitro Test Systems

Cell line CT26 is a mouse colon carcinoma (ATCC collection). СТ26 cells were cultured in RPMI1640 medium (PanEco, Moscow, Russia) supplemented with 2 mM-Lglutamine (PanEco, Moscow, Russia) and 10% fetal bovine serum (FBS) (Biowest, SouthAmerica); in 25 cm2 culture flasks (SPL Life Sciences, Pochon, Republic of Korea) at 37 ◦C in a humid atmosphere with 5% carbon dioxide. Cells were subcultured two times per week. During passaging, Versene solution (PanEco, Moscow, Russia) was used to remove cells from the substrate. For in vitro studies, cells of the 3rd to 5th passages with an optimal seed concentration.

### Confocal microscopy

CT26 cells were seeded in a Petri dish in 1.5 mL of growth medium (200 × 10^3^ cells/well) and cultured for 24 h. After this, the nutrient medium was removed and replaced substances in PBS. Cells were incubated at different times (15, 30, 60 and 120 min) and twice washed with HBSS (with calcium and magnesium ions). Cells were visualized with using a Nikon Eclipse Ti2 microscope (Nikon, Tokyo, Japan) equipped with a laser scanning (ThorLabs, Newton, New Jersey, USA) and water immersion Apo 25 ×/1.1 lenses. Scanning was carried out using software ThorImageLS software (version 2.4) (Thorlabs, Newton, NJ, USA); for processing Fiji software was used for images.

### Phototoxicity

To study photoinduced and cytotoxic activity, tumor cells were seeded in 96-well plates in an amount of 10 × 10^3^ cells per well in 100 µL of complete growth medium. The cells were exposed to the exponential phase of cell growth 24 h., after seeding. Solutions of compounds in complete culture medium were added to the wells at a final concentration from 20 ng/mL to 4000 ng/mL, in triplets. The duration of incubation of cells with compounds before irradiation was 0.5, 2 and 4 h. The culture medium was used as a control. Light exposure was carried out with a halogen lamp through a KS-10 broadband filter (λ > 620 nm). The light dose was 10 J/cm^2^. After completion of light exposure, the plates with cells were placed in a CO_2_ incubator for 24 h. To assess cytotoxicity, cells were incubated with the substances for 24 h., in darkened conditions in a CO_2_ incubator. Cell survival was assessed using the colorimetric MTT test (24 h., after irradiation), which is based on the ability of living cell dehydrogenases to convert yellow water-soluble 3-(4–5-dimethyl-tetrazolyl-2)−2,5-diphenyltetrazolium bromide into blue crystals formazan, the amount of which is measured spectrophotometrically.

Before starting the test, the cells were washed from cytotoxic agents, then a 0.5% solution of 3-(4,5-dimethylthiazolyl-2)−2,5 diphenyl tetrazolium bromide was added to the wells and incubated for 3 h., under standard conditions. After completion of incubation, the medium with the MTT reagent was removed and the formazan crystals were dissolved with dimethyl sulfoxide for 10 min. Optical density was measured on a Multiscan FC multichannel tablet photometer (Thermo scientific) at a wavelength of 550 nm.

The level of growth inhibition (GI) of cells in culture was calculated using the formula:

$${\text{GI }}\left( \% \right){\text{ }}={\text{ }}[{\mathrm{O}}{{\mathrm{D}}_{\mathrm{k}}}-{\mathrm{O}}{{\mathrm{D}}_{\mathrm{o}}}/{\mathrm{O}}{{\mathrm{D}}_{\mathrm{k}}})]{\text{ }}^*{\text{ }}100\% ,$$

Where OD_o_ is the optical density of formazan in the experimental wells, OD_k_ is the optical density of formazan in the control wells. A biologically significant effect was considered inhibition of cell growth in culture by more than 50% (GI_50_). Quantitative parameters were calculated from three independent tests. Quantitative data were expressed as the mean ± confidence interval.

For ROS quantification. CT26 cells were seeded in a black 24-well plate with a clear bottom (IBIDI, German) and incubated without treatment for 24 h. After that, the cells were washed twice with Hank’s solution, 5 µM H2DCFDA (ThermoFisher, USA) was added, and the cells were incubated for 30 min. Then, the cells were treated with nanoparticle solutions at concentrations corresponding to the IC50, incubated for 30 min, and exposed to light (10 J/sm2). Images were then taken using an EVOS FL epifluorescence microscope (ThermoFisher, USA) using a GFP filter (Sup. Figure 9, 10).

## Results

### Characterization of NPs, NP/DOPAC and NP/DOPAC/PEG

**Fig. 1:**
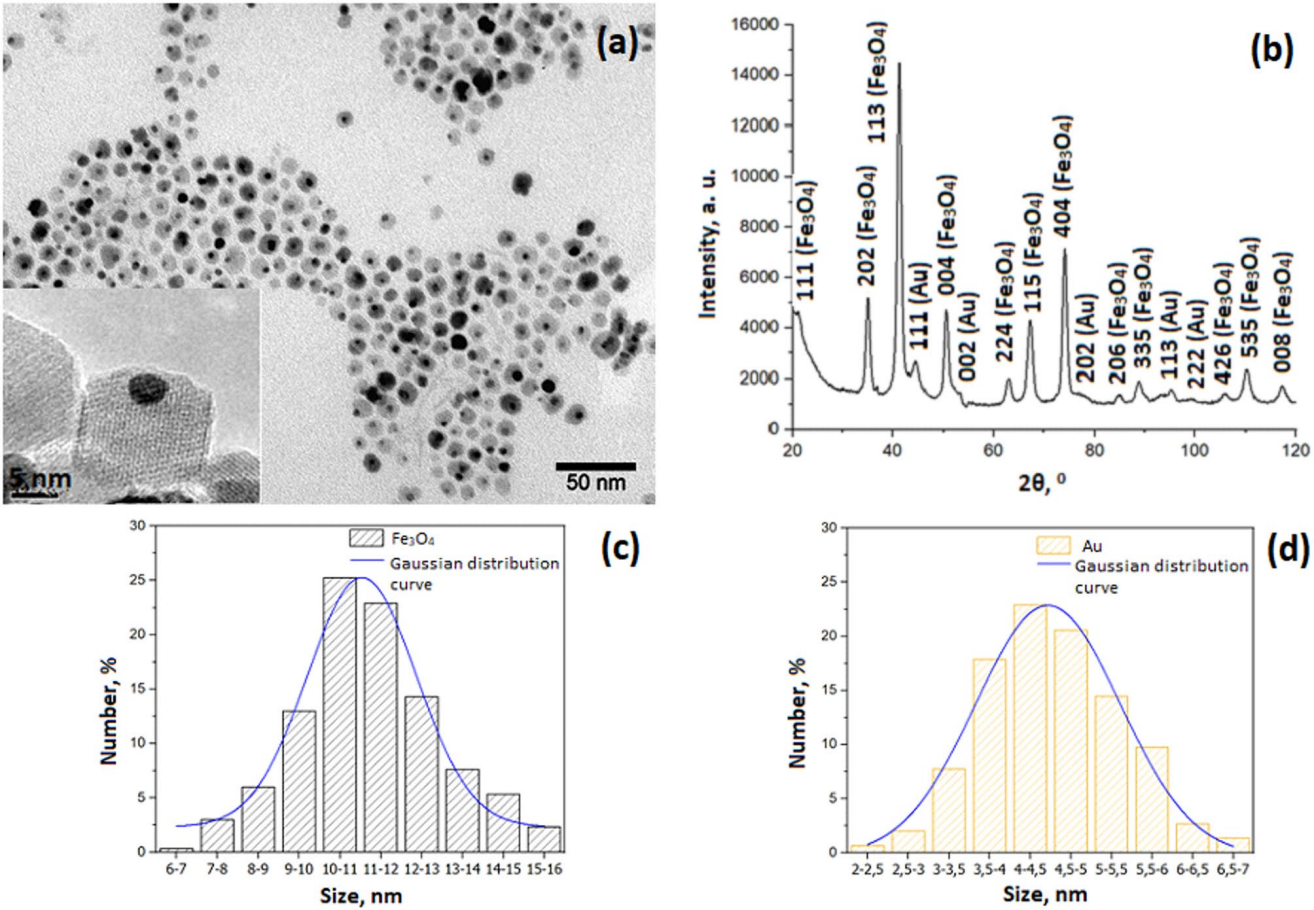
TEM images of dumbbell iron oxide - gold NPs **(a)**; X-ray powder diffraction of NP **(b)** and size distribution of iron oxide and gold NPs (c, d).

Synthesized NPs consist of magnetite and gold NPs with sizes of 11.2 ± 1.5 nm and 4.4 ± 1.0 nm for magnetite and gold, respectively, measured by TEM (Fig. 1a). The presented micrographs (TEM) (Fig. 1a) show gold NPs (dark) and magnetite NPs (lighter) connected in pairs. In addition, it can be noted that the resulting sample does not contain aggregates of NPs, as well as free NPs of magnetite or gold. Such sizes suggest minimizing the FRET effect between PS and FP, since the calculated parameter is the Förster radius for this chromophore pair was 15 Å^27^. There is also a good agreement between the average crystallite size according to XRD data and the average diameter of NPs according to TEM data. We can also state that the synthesized NPs are monodisperse (Fig. 1c and d). In addition, according to the X-ray diffraction patterns (Fig. 1b), there is a 100% correspondence between the spectra of the magnetite (ICDD PDF-2 No. 00–019-0629) and gold (ICDD PDF-2 No. 03–065-8601) components of all samples of Fe_3_O_4_-Au NPs, where the type of structure of cubic spinel Fd-3 m (magnetite-type structure) was determined (Table 1).

**Table 1:** NP TEM and X-ray Powder Diffraction data.

TEM			X-ray Powder Diffraction					
d, nm		particle shape	volume fraction, %		crystallite size, nm		lattice period, nm	
Fe_3_O_4_	Au		Fe_3_O_4_	Au	Fe_3_O_4_	Au	Fe_3_O_4_	Au
11.2 ± 1.5	4.4 ± 1.0	spherical	94.0 ± 2.0	6.0 ± 2.0	12.0 ± 2.0	5.1 ± 1.0	0.8387 ± 0.0004	0.4064 ± 0.0004

**Fig. 2:**
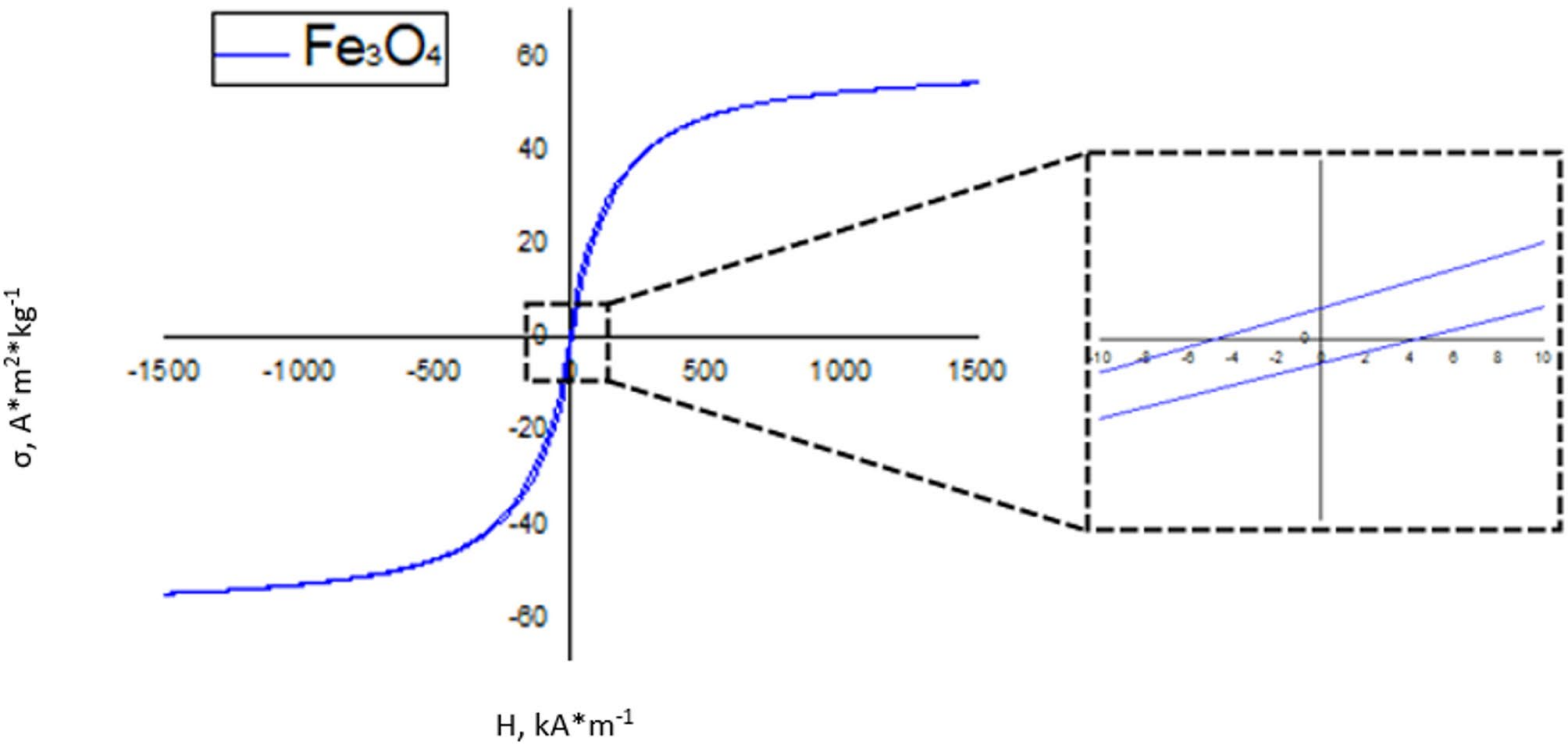
Magnetization curve of Fe_3_O_4_ – Au NPs of the “dumbbell” type.

The magnetometry results (Fig. 2), where the specific saturation magnetization is equal to 55.2 (σ_s_, A·m^2^·kg^− 1^), the residual saturation magnetization is equal to 1.3 (σ_r_, A·m^2^·kg^− 1^), the coercive force is equal to 3.3 (H_s_, kA·m^− 1^) at room temperature, indicate the superparamagnetic nature of the NP samples (Fe_3_O_4_-Au) (with sizes less than 20 nm), which is typical for magnetite nanonuclei of such sizes and is in good agreement with the literate data. The values ​​of all the obtained parameters (σ_s_, σ_r_, H_s_) correlate with the data described in the literature^28^.

For effective delivery to the target tumor tissue, NPs must ensure colloidal stability in water solutions with pH and ionic strength mimicking conditions of physiological liquids, such as blood. Besides colloidal stability also long-term circulation in blood stream is required to achieve effective delivery of NPs to tumor. Conventional surface unmodified NPs are usually captured by reticuloendothelial system (RES), such as liver and spleen^30^. Proposed mechanism of NP elimination from blood stream includes interaction with residential macrophages of RES organs, such as liver and spleen. Two main factors regulating NPs interaction with macrophages are size and surface coating^31^. It was shown that increase in size correlates with increase of NP uptake by macrophages, leading to decrease in circulation time^32^. It was shown that critical size for NPs is somewhere around 100 nm^33^. For NPs with size above this value significant drop-in blood circulation time is usually observed, especially for magnetic NPs. For NPs with size below 100 nm no such dramatic changes are observed, however there is still observed tendency in increase of blood circulation time with decrease of size of NPs up to tens of nanometers^34^. Second factor is chemical structure of NPs surface. Unmodified NPs are hydrophobic and easily can interact with plasma proteins in process called opsonization^35^. Appearance of plasma proteins on surface of NPs significantly increase their uptake by macrophages, thus reducing time of circulation^36^. One of the most popular approaches to reduce opsonization of NPs is modification of NPs surface by polyethylene glycol (PEG). PEG molecules create non charged hydrophilic layer, which prevents plasma proteins from interaction with NPs surface, thus preventing opsonization^37^. As seen from Table 2the sizes of all NP is less than 30 nm with negative surface charge less than − 20 mV, as measured by DLS technique. Making it possible to achieve long-circulation in the blood, which will increase the efficiency of passive targeting. It is worth mentioning the additional modification of the surface of NPs with DOPAC and PEG (Mn 1.100), which helps to reduce the hydrophobicity a of the system, and also might increase the circulation time in the blood, reduce the capture by macrophages and protect against opsonization by repelling plasma proteins^33,38^.

**Table 2:** Size-distribution and ζ-potential of systems.

	NP/DOPAC	NP/DOPAC/PEG	NP/PS	NP/FP	NP/PS/FP
Hydrodynamic size, nm	17.1 ± 1.5	18.3 ± 1.4	20.5 ± 2.0	21.5 ± 2.0	25.9 ± 2.1
PDI	0.381	0.345	0.377	0.275	0.299
ζ-potential, mV	−24.0 ± 2.0	−26.3 ± 2.0	−20.4 ± 2.0	−36.6 ± 3.0	−25.5 ± 2.0

On each step of modification hydrodynamic diameters of NP were measured by DLS technique. The hydrodynamic diameter of the NPs increased, after each modification step which proves the formation of additional organic layers of DOPAC, PEG, FP and PS consequently. Also, it is seen that after hydrophilization of NP by formation of DOPAC layer on the surface of the magnetic cores of the NPs due to the formation of Fe-O bonds with the hydroxyl groups of DOPAC the overall size of NP is only slightly increased by 1–2 nm, indicating that such coating allows not only effective phase transfer from organic solvents, but prevents NP from aggregation into a structures, where more than one NP is embedded into the DOPAC shell. We believe that this is an important issue, because during aggregation of NP inside DOPAC orientation of Au and Fe_3_O_4_ surfaces becomes unpredictable leading to situation where these surfaces will be oriented close one two another, thus allowing PS and FP approach at a distances short enough for FRET to occur, which is strongly undesirable.

### Immobilization of chromophores on the surface of NPs

After the stable water solutions of DOPAC-PEG coated NP were obtained another problem that was needed to be solved is selective immobilization of PS onto the surface of DOPAC-PEG coated magnetite. The main problem arises from poor solubility of PS in water not only preventing its conjugation to NP/DOPAC/PEG but also stacking of the PS molecule is observed in water, which leads to the loss of ROS production and fluorescence (Sup. Figure 8)^39^. On the other hand, high amount of organic solvent leads to loss of colloidal stability of NP/DOPAC/PEG.

In our previous work, it was shown that proper solvent could be obtained by use of DMSO/water mixture, where at certain DMSO/water ratio PS retains its phototoxicity and fluorescence and NP/DOPAC/PEG solution is still colloidally stable^40^. Based on evaluation of PS fluorescence intensity in different DMSO/water ration PS immobilization was carried out in a DMSO/H_2_O mixture with a ratio of 30/70 by volume. At this DMSO/water volume ratio PS still retains its photoactivity and NP/DOPAC/PEG do not aggregate. Immobilization was carried out to create NP/PS and NP/PS/FP systems with preliminary activation of the carboxyl group of PEG by the carbodiimide method. The loading of PS on the magnetic surface of NPs was calculated, which varied in the range of 38–40%. For the conjugation of FP to the gold surface of NP/DOPAC/PEG, a thiol or disulfide group is required to form a strong (with an energy of 40 kcal/mol) covalent Au-S bond. The disulfide form of the dye was synthesized according to the method in from Cy5 sulfo NHS ester (*Sup. Figure 3a*). FP loading on the gold NP surface was calculated, which varied in the range of 20–25%^41^.

**Fig. 3:**
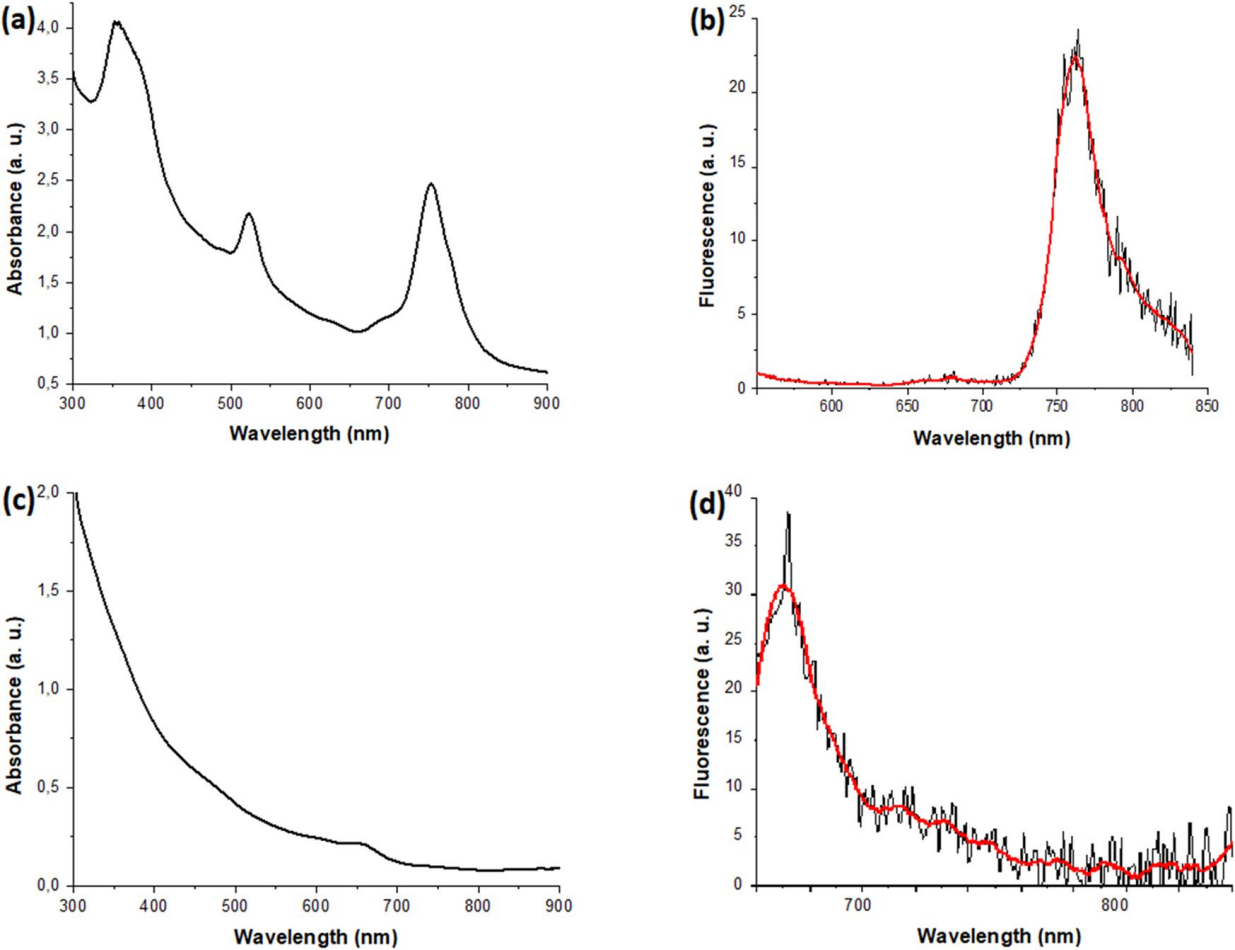
**(a)** absorbtion spectra of NP/PS; **(b)** fluorescence spectra of NP/PS (excitation wavelength = 520 nm); **(c)** absorbtion spectra of NP/FP; **(d)** fluorescence spectra of NP/FP (excitation wavelength = 650 nm).

**Fig. 4:**
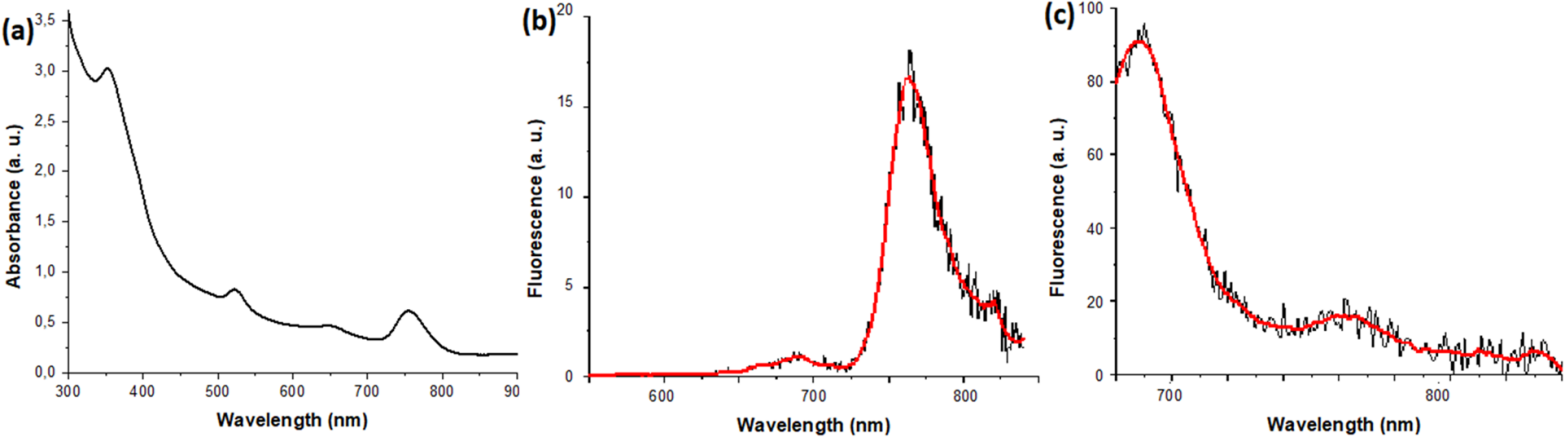
**(a)** absorption spectra of NP/PS/FP; **(b)** fluorescence spectra of NP/PS/FP (excitation wavelength = 520 nm); **(c)** fluorescence spectra of NP/PS/FP (excitation wavelength = 650 nm).

We found an overall increase in NP size after covalent loading for both types of chromophores. We believe that due to the immobilization of PS and FP on the surface of NPs, increased hydrophobicity of the outer shell occurs, which can lead to an increase in the size of the coating. In addition, after loading, absorption peaks of PS at 530 and 760 nm (Fig. 3a), and an emission peak of 760 nm (excitation 530 nm) were detected in the NP/PS (Fig. 3b) and NP/PS/FP (Fig. 4b) systems, which confirms the presence of PS on the surface of NPs. The absorption peak at 650 nm (Fig. 3c) and the emission peak at 680 nm (excitation 650 nm) confirm the efficient conjugation of FP onto the gold NP surface in the NP/FP (Fig. 3d) and NP/PS/FP (Fig. 4c) systems. We can also see a broad absorption peak at 350 nm, especially in the region below 350 nm, which is typical for magnetite NP cores. In the Fig. 4, a, we observe all four absorption peaks for the NP/PS/FP system corresponding to the absorption peaks of PS and FP, which shows effective immobilization of both PS and FP. Moreover, when the NP/PS/FP system is excited by wavelength of 530 nm (PS excitation) (Fig. 4b), we observe only PS fluorescence, however, when exposed to 650 nm (FP excitation) (Fig. 4c), additional emission is visible FS in the region of 770 nm, associated with energy transfer via the FRET mechanism from the energy acceptor (FP) to the donor (PS)^42^. The energy transfer exhibited at a relatively low intensity was expected. Since dimeric NPs have a “dumbbell” shape, at the junction of two spherical surfaces (magnetite and gold) there will always be sterically close molecules of PS and FP. It is important to understand that the number of such molecules with an intermolecular distance less than the Förster radius is extremely small, which is what we observe in the form of a low-intensity peak that will not affect the therapeutic effectiveness of the platform.

It can be noted that the peak of absorption of PS at IR region of optical spectra is clearly seen. This fact is important because it is known that human body is mostly transparent in IR region, thus allowing effective light penetration of tissue in biological window, which is important for deeper located tumors therapy^43,44^. In addition, irradiation by light have lower risk of tissue over-heating in near-infrared area^45,46^.

### Study of cytotoxicity, phototoxicity and internalization of systems by the CT26 cell line

#### Cytotoxicity and phototoxicity of systems

**Fig. 5:**
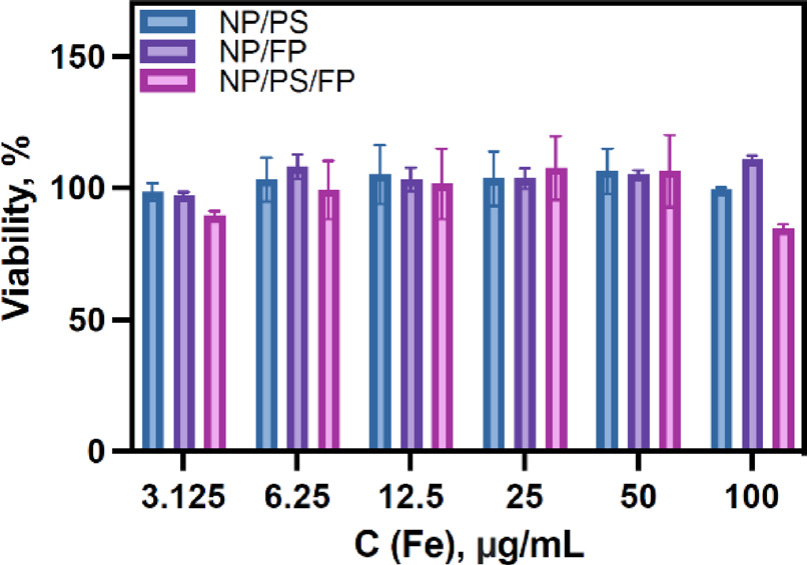
Cytotoxicity data (CT26 cell line) NP/PS, NP/FP and NP/PS/FP.

Toxicity is one of the most important elements in the biological applications of nanomaterials. Toxicity of all three types of NP were evaluated by MTT test on CT26 cell culture in the studied concentration range (C_Fe_ from 3.125 to 100 µg/mL) (Fig. 5). We did not observe any significant cytotoxic effects from all three types of NP except NP/PS/FP, whereat concentration 100 mg/mL, a slight decrease (about 20%) in viability was observed.

Being not toxic to cell lines after simple incubation is obligatory, but does not correlate with ability of NP induce cell death under light irradiation. The photoinduced toxicity of the NP/PS, NP/FP, and NP/PS/FP (Table 3) systems was studied also on the СT26 tumor cell line at the concentration of Fe in all systems equal to 1.5–1.6 mg/mL, corresponding to concentration of PS in systems equal to 0.25 mg/mL. As expected, the NP/FP system without a PS does not exhibit pronounced phototoxicity, GI50 > 4000 ng/mL over 4 h of incubation.

For the NP/PS and NP/PS/FP photoinduced cytotoxicity was observed. Photoinduced cytotoxic effect was increased with increase of incubation time from 30 min to 4 h, which we explain by higher uptake efficiency of NP by cells. Interestingly, we observed significant increase of NP/PS/FP photoinduced cytotoxicity in comparison to NP/FP system, despite of the fact that concentrations of FP were equal. We find an explanation of this fact in fluorescence spectra of both systems. As was previously show on Figs. 3d and 4c for NP/PS/FP additional peak of PS fluorescence is observed at 750–770 nm range, indicating that there is small fraction of PS and FP close one to another allowing FRET to occur and transfer energy from FP to PS, thus increasing it is fluorescence and ROS production. As described in Materials and Methods section for photoinduced cytotoxicity we used a filter with λ > 620 nm, meaning that FP will also absorb the light at this wavelength, allowing FP to be excited and transfer an energy to PS molecules, thus increasing overall photoinduced cytotoxicity.

**Table 3:** Photoinduced activity of substances (CT26 cell culture).

GI50 (ng/mL)			
System	Incubation time, h		
	0.5	2	4
NP/PS	> 4000	3320 ± 19	1799 ± 14
NP/FP	> 4000	> 4000	> 4000
NP/PS/FP	2318 ± 16	1172 ± 13	483 ± 7

#### Internalization of systems

**Fig. 6:**
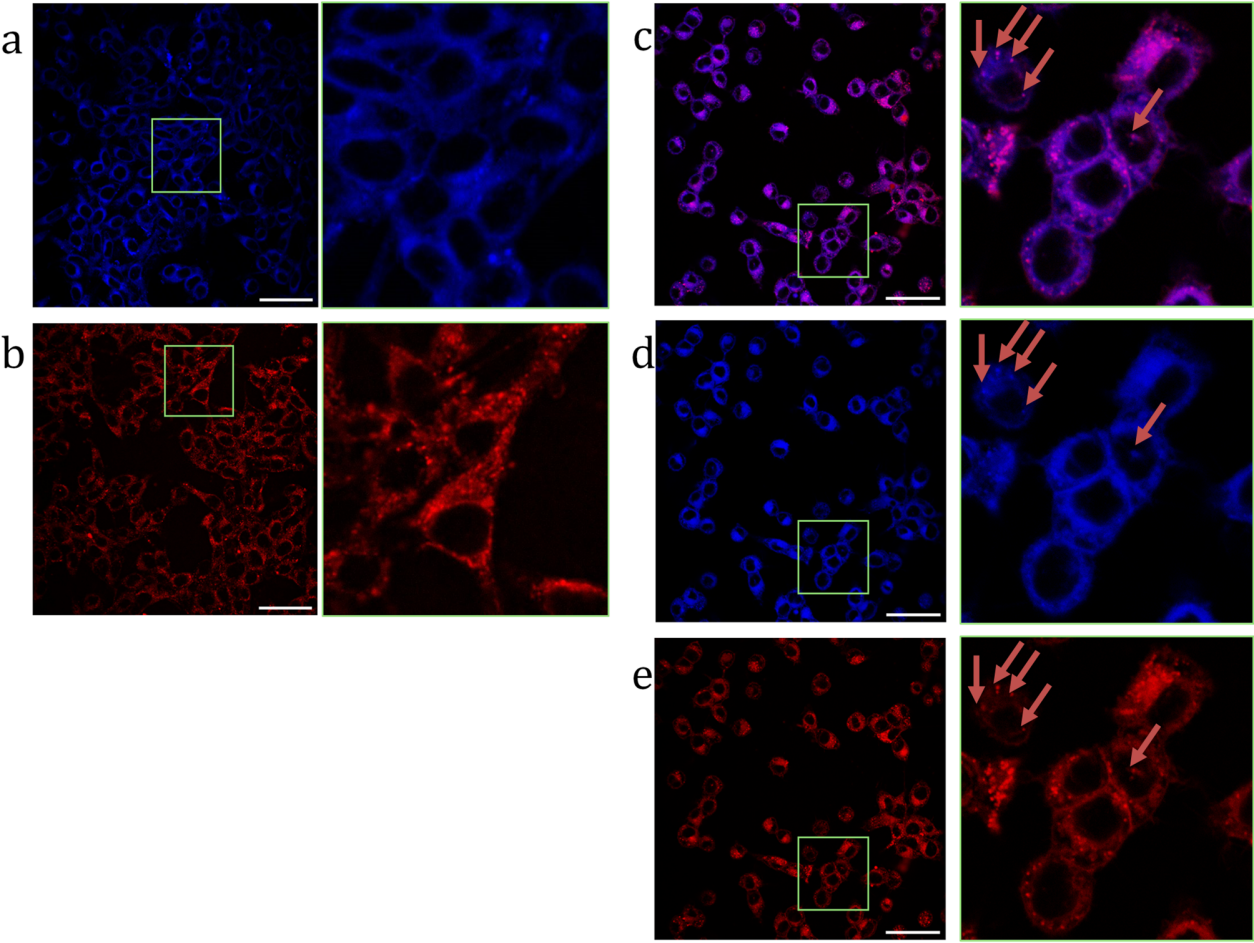
Confocal imaging of CT26 cells incubated for 2 h., with the systems: **(a)** NP/PS; **(b)** NP/FP; **(c)** NP/PS/FP (merge); **(d)** NP/PS/FP (excitation wavelength = 405 nm); **(e)** NP/PS/FP (excitation wavelength = 642 nm); (scale 50 μm).

Based on fluorescence and absorbance measurements we can conclude that our approach allows to separate FP and PS molecules to sufficiently reduce FRET. However, being internalized in cells NP can aggregate or the enzymatic cleavage of bonds connecting FP and PS to NP can occur. For evaluation of this effect cell distribution of three types of NP was evaluated by confocal microscopy. For all three types of NPs, we observed their uptake by CT26 cells, however patterns of their internalization were different. In case of NP/PS and NP/PS/FP the internalization patterns where similar, and what is more important for NP/PS/FP we observed similar colocalization in both fluorescent channels for PS and for FP, indicating that even 2 h after internalization both PS and FP are connected to the NP surface. In case of NP/PS and NP/PS/FP we did not observe nuclear localization. Cytoplasmic or perinuclear localization did not appear to be uniformly distributed, and some more intensely fluorescent spots could be identified (Fig. 6). For NP/FP system we observed less even distribution, and most of signal was detected in the form of small spots, probably corresponding to lysosomes. We believe that this result arises from presence of PS molecules on the surface of NP/PS and NP/PS/FP, thus changing their intracellular distribution in comparison with NP/FP. Also, the presence of PS on surface of NP seemed to be more important factor affecting NP internalization, as we observe that in NP/PS/FP localization of fluorescent signal from FP nicely correlates with localization of fluorescent signal from PS (Fig. 6d) and FP signal in cells incubated with NP/PS/FP do not show the same pattern as observed for NP/FP (Fig. 6b).

Thus, all systems exhibit localized fluorescence in their respective wavelength range and are capable of being internalized in the cytoplasm of cancer cells (Fig. 6a-e). In Fig. 6c shows the colocalization of PS and FP signals, which indicates the presence in the cytoplasm of cells of NPs having covalently conjugated PS and FP on their surface (NP/PS/FP).

## Conclusion

In our work, we showed that dimeric magnetite-gold dumbbell –like nanoparticles could be used as a platform for combining chromophores for the joint use of fluorescence diagnostics and photodynamic therapy, eliminating the FRET effect. Modification of DOPAC and PEG NPs with subsequent activation of EDC/NHS allows carrying out effective conjugation of the PS onto the magnetic surface of NPs in a two-phase system (water-DMSO). Moreover, when loaded onto NPs, PS retain their properties as a phototoxic agent, which makes it possible to use them for further photodynamic therapy. The use of a disulfide derivative of the cyanine dye Cy5, covalently immobilized on the gold surface of NPs, makes it possible to detect the system. It was shown that the NP/PS, NP/FP, and NP/PS/FP systems are capable of being internalized by CT26 colon cancer cells while maintaining optical properties. In addition, the systems do not have dark cytotoxicity up to a concentration of 100 µg/mL. The final NP/PS/FP system exhibits high phototoxicity, which allows planning in vivo studies in the future. It’s worth noting that the use of Fe_3_O_4_nanoparticles in this study allows them to be used not only as a platform for PDT but also for chemodynamic therapy, which could also help improve the effectiveness of theranostics. This opens up several prospects, such as further expansion of research using synergistic therapies^47,48^.

## Supplementary Information

Below is the link to the electronic supplementary material.

Supplementary Material 1

Supplementary Material 2

Supplementary Material 3

Supplementary Material 4

## Data Availability

Data will be made available on request from Iuliia Chudosai.
