# Supplementary material for: Dimeric magnetic dumbbell nanoparticles with selective immobilization of chromophores for improved tumor theranostics
Source: Sci Rep. 2026 Mar 5;16:12101. doi: 10.1038/s41598-026-40586-4 (PMC13076682; doi:10.1038/s41598-026-40586-4)
Supplement: Supplementary file 1 — Supplementary Material 1 [file 41598_2026_40586_MOESM1_ESM.docx]

**Additional Information:**

Selective immobilization of fluorescent dye and photosensitizers on dumbbell like magnetite-gold nanoparticles for improved tumor theranostics.

*Iuliia Chudosai ^1^, Petr Ostroverkhov ^4^, Ekaterina Plotnikova ^4,5^, Kseniya Stepanova ^3^, Nelli Chmelyuk ^2,3^, Elizaveta Ivanova ^2,3^, Mihail A. Grin ^4^, Olga Fedorova ^6^, Natalia Klyachko ^1^, Vladimir P. Chekhonin ^2,3^, Maxim Abakumov ^2,3,*^.*

*^1^* Department of Chemistry, Lomonosov Moscow State University, Moscow 119991, Russia

*^2^* Pirogov Russian National Research Medical University, Ostrovitianov Str. 1, 117997 Moscow, Russia

*^3^* National Research Technological University “MISIS”, Biomedical Nanomaterials, Leninskiy prospekt 4, 119049 Moscow, Russia

*^4^* Russian Technological University (MIREA), 86 Vernadsky Avenue, 119571 Moscow, Russia

*^5^* National Medical Research Radiological Centre of the Ministry of Health of the Russian Federation, P.A. Hertsen Moscow Oncology Research Institute, 125284 Moscow, Russia

*^6^* A. N. Nesmeyanov Institute of Organoelement Compounds, Russian Academy of Sciences, 119334 Moscow, Russian Federation

*^*^* [abakumov1988@gmail.com](mailto:abakumov1988@gmail.com)


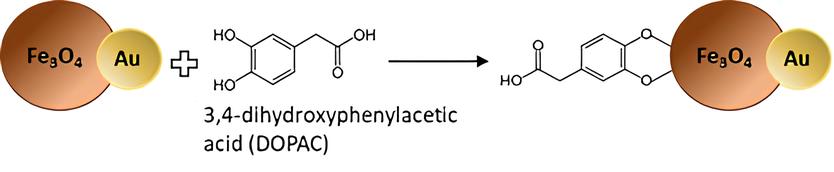


1. Scheme of DOPAC coating with Fe_3_O_4_-Au NPs (NP/DOPAC)


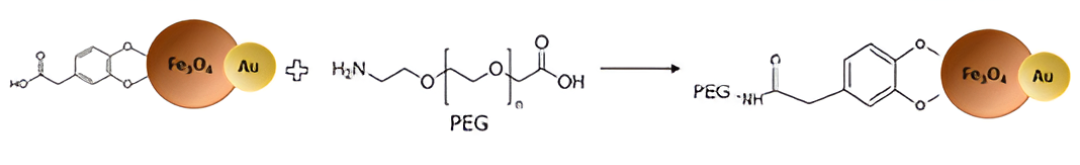


1. Scheme of PEG coating of NP/DOPAC (NP/DOPAC/PEG)


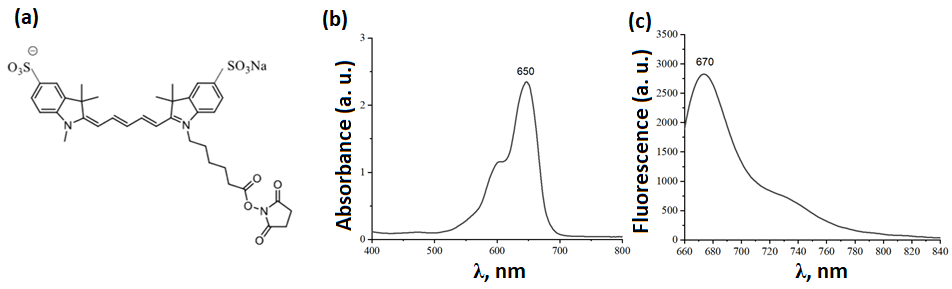


1. (a) structure of FP; (b) absorbtion spectra of FP; (c) fluorescence spectra of FP (excitation wavelength = 650 nm)


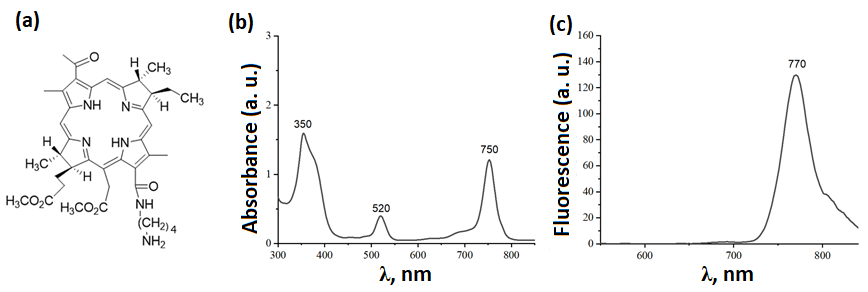


1. (a) structure of PS; (b) absorbtion spectra of PS; (c) fluorescence spectra of PS (excitation wavelength = 520 nm)


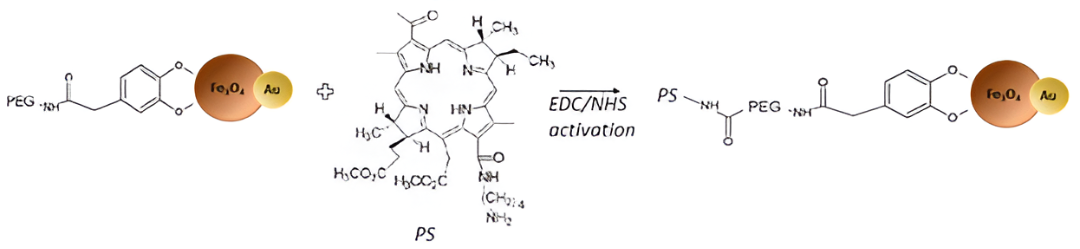


1. Scheme of covalent conjugation of NP/DOPAC/PEG with PS (NP/PS)


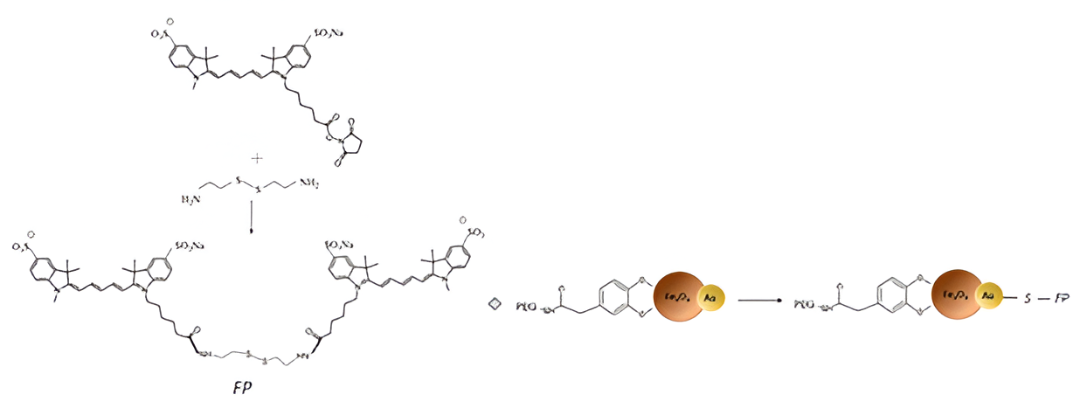


1. Scheme of covalent conjugation of NP/DOPAC/PEG with FP (NP/FP)


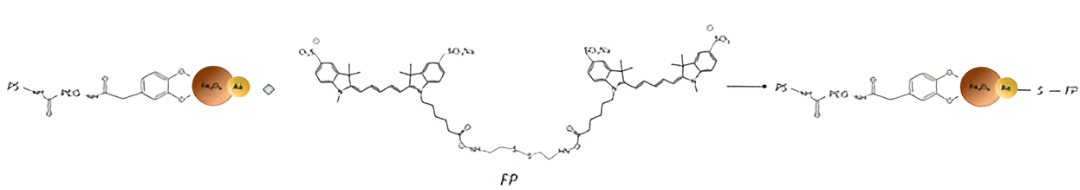


1. NP/PS/FP synthesis scheme


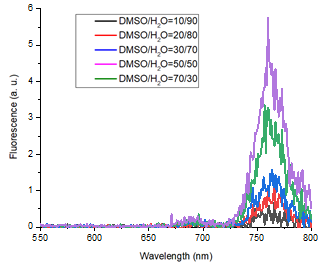


1. Fluorescence spectra of PS in solutions with different DMSO/H_2_O (excitation wavelength 530 nm)
