# Supplementary material for: Dimeric magnetic dumbbell nanoparticles with selective immobilization of chromophores for improved tumor theranostics
Source: Sci Rep. 2026 Mar 5;16:12101. doi: 10.1038/s41598-026-40586-4 (PMC13076682; doi:10.1038/s41598-026-40586-4)
Supplement: Supplementary file 3 — Supplementary Material 3 [file 41598_2026_40586_MOESM3_ESM.docx]

**Supplementary materials:**

Dimeric magnetic dumbbell nanoparticles with selective immobilization of chromophores for improved tumor theranostics.

*Iuliia Chudosai ^1^, Petr Ostroverkhov ^4^, Ekaterina Plotnikova ^4,5^, Kseniya Stepanova ^3^, Nelli Chmelyuk ^2,3^, Elizaveta Ivanova ^2,3^, Mihail A. Grin ^4^, Olga Fedorova ^6^, Natalia Klyachko ^1^, Vladimir P. Chekhonin ^2,3^, Maxim Abakumov ^2,3,*^.*

*^1^* Department of Chemistry, Lomonosov Moscow State University, Moscow 119991, Russia

*^2^* Pirogov Russian National Research Medical University, Ostrovitianov Str. 1, 117997 Moscow, Russia

*^3^* National Research Technological University “MISIS”, Biomedical Nanomaterials, Leninskiy prospekt 4, 119049 Moscow, Russia

*^4^* Russian Technological University (MIREA), 86 Vernadsky Avenue, 119571 Moscow, Russia

*^5^* National Medical Research Radiological Centre of the Ministry of Health of the Russian Federation, P.A. Hertsen Moscow Oncology Research Institute, 125284 Moscow, Russia

*^6^* A. N. Nesmeyanov Institute of Organoelement Compounds, Russian Academy of Sciences, 119334 Moscow, Russian Federation

*^*^* [abakumov1988@gmail.com](mailto:abakumov1988@gmail.com)


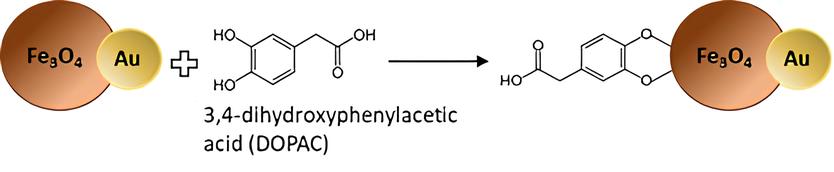


1. Scheme of DOPAC coating with Fe_3_O_4_-Au NPs (NP/DOPAC)


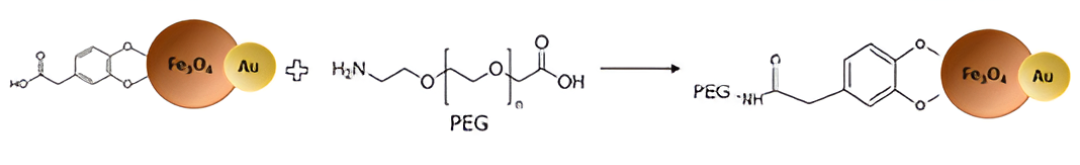


1. Scheme of PEG coating of NP/DOPAC (NP/DOPAC/PEG)


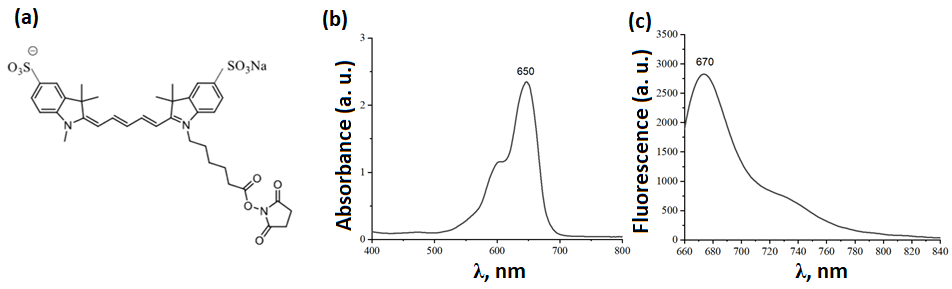


1. (a) structure of FP; (b) absorbtion spectra of FP; (c) fluorescence spectra of FP (excitation wavelength = 650 nm)


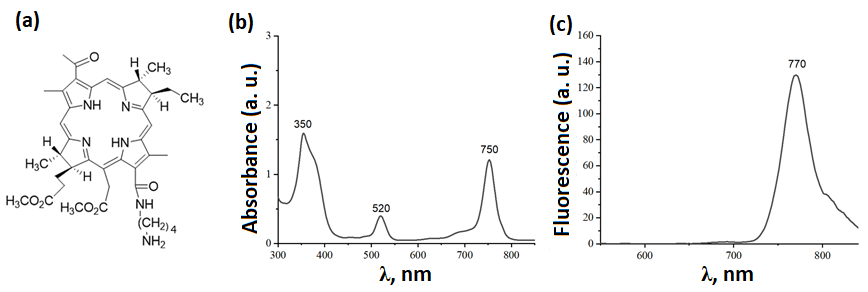


1. (a) structure of PS; (b) absorbtion spectra of PS; (c) fluorescence spectra of PS (excitation wavelength = 520 nm)


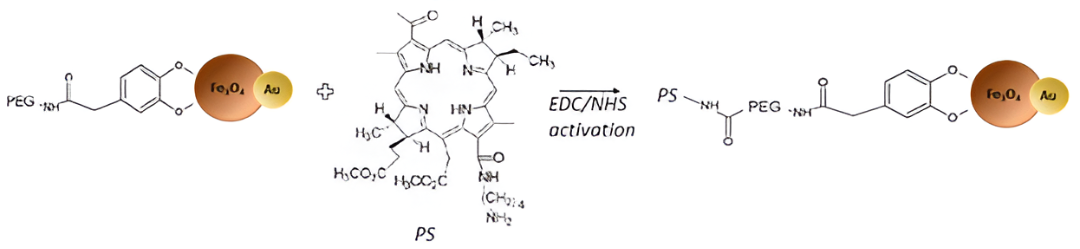


1. Scheme of covalent conjugation of NP/DOPAC/PEG with PS (NP/PS)


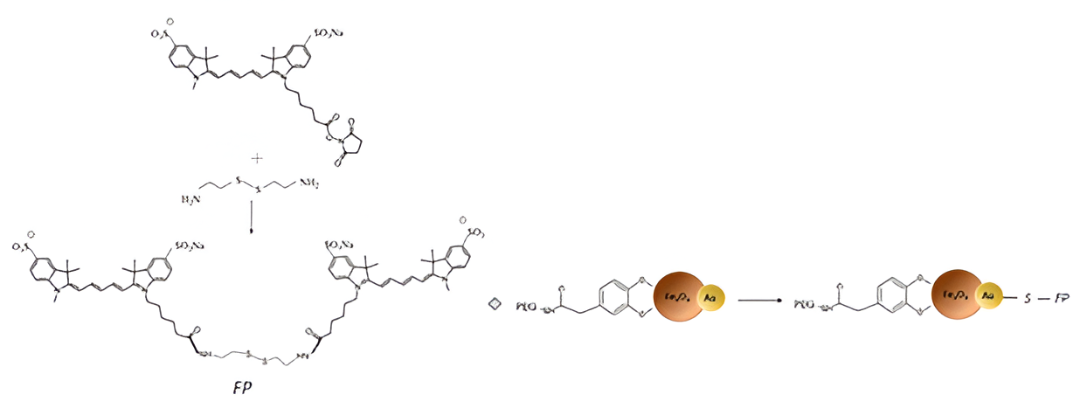


1. Scheme of covalent conjugation of NP/DOPAC/PEG with FP (NP/FP)


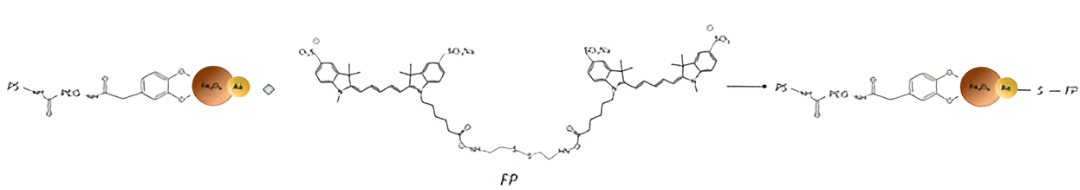


1. NP/PS/FP synthesis scheme


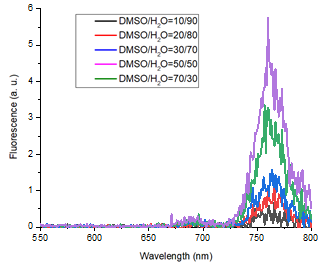


1. Fluorescence spectra of PS in solutions with different DMSO/H_2_O (excitation wavelength 530 nm)


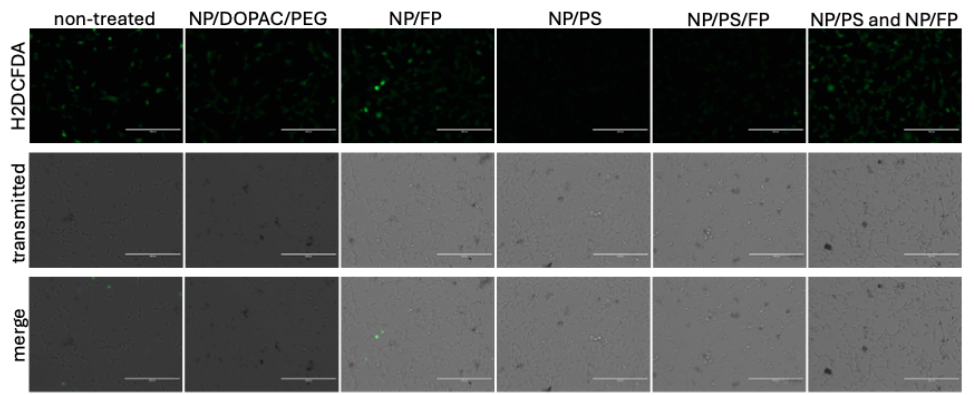


1. Intracellular ROS levels determined using an H2DCFDA ROS probe. Representative fluorescent microscopy images showing intracellular ROS in CT26 after incubation with nanoparticles. Scale bar 200 um.


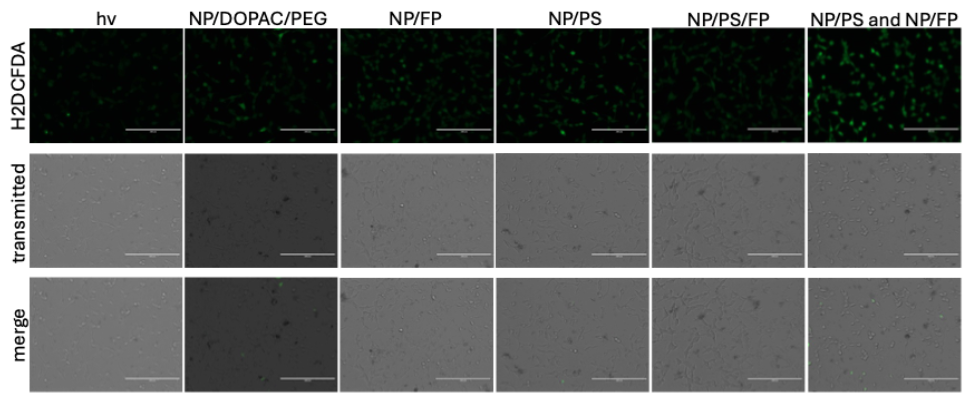


1. Intracellular ROS levels determined using an H2DCFDA ROS probe. Representative fluorescent microscopy images showing intracellular ROS in CT26 after incubation with nanoparticles and light irradiation. Scale bar 200 um.

CT26 cells were seeded in a black 24-well plate with a clear bottom (IBIDI, German) and incubated without treatment for 24 hours. After that, the cells were washed twice with Hank's solution, 5 μM H2DCFDA (ThermoFisher, USA) was added, and the cells were incubated for 30 minutes. Then, the cells were treated with nanoparticle solutions at concentrations corresponding to the IC50, incubated for 30 minutes, and exposed to light (10 J/sm^2^). Images were then taken using an EVOS FL epifluorescence microscope (ThermoFisher, USA) using a GFP filter.
